# Easy Proteomics Sample Preparation: Technical Repeatability and Workflow Optimization Across 8 Biological Matrices in a New Core Facility Setting

**DOI:** 10.1002/pmic.70064

**Published:** 2025-10-24

**Authors:** Paraskevi Karousi, Maria Voumvouraki, Panagiota Efstathia Nikolaou, Ioannis Kollias, Foteini Paradeisi, Elena Sampanai, Vasiliki Gkalea, Ioannis Morianos, Jerome Zoidakis, Efstathios Kastritis, Nikolaos Thomaidis, Guillaume Médard, Julie Courraud

**Affiliations:** ^1^ Section of Biochemistry and Molecular Biology Department of Biology School of Science National and Kapodistrian University of Athens Athens Greece; ^2^ Proteomics Core Facility, School of Science National and Kapodistrian University of Athens Athens Greece; ^3^ Laboratory of Analytical Chemistry, Department of Chemistry, School of Science National & Kapodistrian University of Athens Athens Greece; ^4^ Laboratory of Pharmacology Department of Pharmacy School of Health Sciences National & Kapodistrian University of Athens Athens Greece; ^5^ Section of Clinical Therapeutics Department of Medicine School of Health Sciences National and Kapodistrian University of Athens Athens Greece; ^6^ Proteomics Laboratory, Biomedical Research Foundation Academy of Athens Athens Greece; ^7^ Hematology Department Alexandra General Hospital Athens Greece; ^8^ Host Defense & Fungal Pathogenesis Lab Institute of Molecular Biology and Biotechnology, Foundation for Research and Technology Athens Greece

**Keywords:** bottom‐up proteomics, diaPASEF, SPEED protocol, tryptic peptides, technical repeatability

## Abstract

Bottom‐up proteomics relies on efficient and repeatable sample preparation for accurate protein identification and precise quantification. This study evaluates the performance of adapted SPEED (Sample Preparation by Easy Extraction and Digestion) protocol, a simplified, detergent‐free approach tailored for various biological matrices, including lysis‐resistant samples. Protein extraction and denaturation steps were refined for 8 biological matrices enabling standardized, cheap, and scalable proteomics analysis on 96‐well plates. For tissue samples requiring downstream applications like Western blotting, we used a low‐detergent RIPA buffer. Notably, the protocols demonstrate remarkable down‐scalability, enabling robust proteomics measurements from as few as 3000 cells per sample for preparation and even down to 300 cells per LC‐MS/MS analysis. Key advancements include a 30‐min nanoLC‐MS/MS run, achieving a 15–20 samples‐per‐day throughput, and leveraging the power of diaPASEF using thoroughly optimized DIA‐windows to enhance proteome coverage. These adaptations streamline workflows, enabling proteomics analyses in matrices with challenging physical and biochemical properties. This study underscores the importance of early‐stage optimization and feasibility testing in proteomics pipelines to inform study design and sample selection. By showcasing robust, scalable adaptations of the SPEED protocol, we provide a foundation for reproducible, high‐throughput proteomic studies across diverse biological contexts.

**Abbreviations**
AcOH: Acetic acid
BAL: BronchoAlveolar Lavage
CV: Coefficient of Variation
DIA: Data‐Independent Acquisition
diaPASEF: Data‐Independent Acquisition‐Parallel Accumulation Serial Fragmentation
ESI: ElectroSpray Ionization
FACS: Fluorescence‐Activated Cell Sorting
FDR: False Discovery Rate
LC‐MS: Liquid Chromatography‐Mass Spectrometry
MeCN: Acetonitrile
RIPA: RadioImmunoPrecipitation Assay
SP3: Single‐Pot, Solid‐Phase‐enhanced Sample Preparation.
SPEED: Sample Preparation by Easy Extraction and Digestion
TFA: TriFluoroAcetic acid
timsTOF: Trapped Ion Mobility Spectrometry Time‐Of‐Flight
tris: Tris(hydroxymethyl)aminomethane

1

Bottom‐up proteomics, where proteins are enzymatically digested into peptides for mass spectrometric analysis, is one of the most widely used techniques for protein identification and quantification. However, the success of this approach is heavily influenced by the efficiency, scalability, and repeatability of the sample preparation process, as well as by the early assessment of technical variability to inform appropriate study designs. At the National and Kapodistrian University of Athens, we set up a new Proteomics Core Facility, facing the challenge (and opportunity) to implement sample preparation pipelines for over 50 projects within the last 2 years. Therefore, we chose a versatile approach to simplify method development across 8 different biological matrices, quickly providing cheap, easy, and fast sample preparation workflows, while ensuring down‐scalability, without compromising  proteome coverage or robustness.

One of the primary challenges in bottom‐up proteomics is the effective extraction of proteins while avoiding detergents and chaotropic agents, which, though useful for extraction, interfere with enzymatic digestion and liquid chromatography‐mass spectrometry (LC‐MS) analysis [1]. The SPEED (Sample Preparation by Easy Extraction and Digestion) protocol offers a simplified, detergent‐free alternative that has proven effective across a wide range of biological matrices [2, 3, 4]. By employing a streamlined three‐step process of acid‐driven lysis, neutralization, and digestion, SPEED enables rapid, efficient, and highly reproducible proteomics sample preparation. This protocol is especially suited for diverse and lysis‐resistant samples, providing consistent results while eliminating the need for detergents. Protein extraction from tissues presents a significant challenge due to their heterogeneity and the dynamic range of the proteome. The extraction method is critical, often requiring specific considerations for compatibility with techniques like Western blotting. The RadioImmunoPrecipitation Assay (RIPA) buffer is well‐established for robust protein extraction while retaining epitope integrity, making it suitable for workflows requiring both proteomic analysis and immunodetection. In this study, we used a low‐detergent RIPA method for tissues (Table S1), alongside SPEED for other matrices, to ensure optimal extraction while addressing the specific needs of each sample type. The aim of this study was to assess and discuss these workflows applied across various biological matrices with differing protein compositions and lysis resistances. Additionally, we emphasize the importance of evaluating technical repeatability in proteomics to assess the feasibility and reliability of the chosen sample preparation methods and to guide study design from the outset.

Tested biological matrices and protocol details are presented in Table 1. The sample types included human platelets, dental plaque, and saliva. Additionally, we tested CD138+ cells isolated from human bone marrow, either as directly analyzed or as protein phenol phase samples after RNA extraction [PPP; inspired by [5]]. Human samples were collected after informed written consent from the donors. Murine heart tissues, murine bronchoalveolar lavage (BAL) samples, and plant‐based samples such as *Cistus creticus* hard and soft seeds were also included. All animal procedures conformed to the Greek Presidential Decree 56/2013 for the protection of the animals used for scientific purposes, in accordance with the European Directive 2010/63/EU. The animal tissue collection was performed as part of the experimental protocols approved by the competent Veterinary Service of the Prefecture of Athens (License protocol numbers 862879/12‐09‐2022 and 179489/13‐02‐2023). All samples were analyzed in three to four replicates. While all these technical replicates were performed in microfuge tubes, the presented protocols are routinely performed on 96‐well plates from the digestion step onwards, minimizing the batch effect for studies encompassing more than 24 samples.

**TABLE 1:** Sample preparation workflows and adaptations for bottom‐up proteomics across various biological matrices, detailing key steps from lysis to nLC‐MS/MS analysis.

	Matrices							
	*Cistus creticus* seeds	Human CD138+ cells from bone marrow	Human CD138+ cells from bone marrow: precipitated protein from RNA extraction	Human dental plaque	Human platelets	Human saliva	Murine bronchoalveolar lavage fluid	Murine heart tissues
Number of replicates	3	3	3	3	4	3	3	3
Minimum initial quantity required	5 mg pulverized seed	30K cells (3K possible)	30K cells	NA	100K	100 µL	4 µg	1 mg
Protein measurement	No	No	NA	No	No	No	Bradford	Lowry
Precipitation & pelleting	No	No	3 volumes acetone	No	No	6 volumes acetone	6 volumes MeCN /acetone	No
TFA lysis	40 µL	Various volumes (cell count‐dependent)	Various volumes (cell counts dependent)	20 µL	120 µL	30 µL	10 µL	8 µL RIPA buffer /mg tissue
Sonication and filtration	10 min	No	No	10 min	No	No	No	No
TFA lysate transfer	No	Various volumes (cell count‐dependent)	Various volumes (cell count‐dependent)	10 µL	1 µL	No	No	1 µL
Neutralization (tris base 2 M)	400 µL	90 µL	90 µL	90 µL	8 µL	300 µL	100 µL	No
Protein measurement	Lowry	No	No	No	Nanodrop—quawell	Lowry	No	Lowry & nanodrop
Neutralized lysate transfer	Various volumes (prot. conc‐dependent)	No	Various volumes (cell count‐dependent)	No	Various volumes (prot. conc‐dependent)	Various volumes (prot. conc‐dependent)	No	Various volumes (prot. conc‐dependent)
Target quantity to digest	4 µg	30K cells	30K cells	NA	2 µg	4 µg	4 µg	4 µg
Volume expansion with tris base 0.1 M	Up to 50 µL	No	No	No	Up to 50 µL	Up to 50 µL	No	110 µL
Reduction/alkylation buffer	5.5 µL	9 µL	9 µL	11 µL	5 µL	5.5 µL	11 µL	11 µL
Heating on the thermomixer	95°C/5 min							
Dilution of tris base with H_2_O	No	360 µL	360 µL	360 µL	No	No	360 µL	No
Trypsin solution (20 ng/µL)	2 µL	2 µL	2 µL	2 µL	1.5 µL	2 µL	2 µL	2 µL
TFA for acidification of the samples before loading	1 µL	Various volumes (cell count‐dependent)	Various volumes (cell count‐dependent)	8 µL	2 µL	2 µL	8 µL	2 µL
Reconstitution with A‐solvent	40 µL	20 µL	20 µL	10 µL	20 µL	40 µL	40 µL	40 µL
Injection volume in the LC‐MS/MS	1 µL	3 µL	3 µL	1 µL	Various volumes (prot. conc‐dependent)	1 µL	1 µL	Various volumes (prot. conc‐dependent)
Proteome(s) used as reference (UniProt ID)	UP000239757	UP000005640	UP000005640	UP000005640; UP000219673; UP001163401; UP001248054; UP000002521; UP000075442; UP000234767; UP000268658; UP000278609; UP000217683; UP000732257; UP001241608; UP001251823; UP000010295; UP000217431; UP000250032; UP000277597	UP000005640	UP000005640; UP000219673; UP001163401; UP001248054; UP000002521; UP000075442; UP000234767; UP000268658; UP000278609; UP000217683; UP000732257; UP001241608; UP001251823; UP000010295; UP000217431; UP000250032; UP000277597	UP000007604	UP000007604

To support reproducible proteomics across diverse sample types, we applied a unified baseline workflow to a broad range of biological matrices. Where necessary, minor modifications were introduced to accommodate distinct sample characteristics. For liquid and non‐viscous samples, total protein content is first estimated using the Lowry [6] or Bradford assay or in some cases with a Nanodrop device (Quawell), to guide downstream processing. If precipitation is required, proteins are precipitated using organic solvents [acetone or acetonitrile (MeCN)] by incubating the mixture at 4°C for 1 h, followed by centrifugation at 18,200 RCF for 5 min at 4°C. The resulting protein pellets are then denatured with trifluoroacetic acid (TFA) and neutralized with 6–8 volumes of tris base 2 M. For solid samples, direct lysis is performed using TFA, except for tissue samples, which are lysed using a low‐detergent RIPA buffer. In cases where lysis with TFA alone does not result in complete sample homogenization, sonication is used post‐lysis to enhance sample disruption and protein extraction. TFA lysates are subsequently neutralized with 6–8 volumes of tris base 2 M. After neutralization (or directly after RIPA lysis), protein content is measured to determine the amount to be digested for these solid samples, unless the input is based on a known cell count, in which case quantification can be omitted. A defined quantity of input is transferred for digestion: 1–4 µg of protein, or an equivalent of 30,000 cells. For all matrices, whenever needed, the volume is adjusted to at least 50 µL using 0.1 M tris base to ensure optimal buffer conditions for enzymatic digestion and enough volume for the reactions. Reduction and alkylation are then carried out by adding 10% volume of a combined tris(2‐carboxyethyl)phosphine hydrochloride (TCEP.HCl, 100 mM) and 2‐chloroacetamide (CAA, 400 mM) solution, followed by vortexing and heating at 95°C for 5 min with shaking at 400 rpm. The concentration of tris base is reduced to less than 0.5 M by adding water (if necessary), ensuring the pH is maintained between 8 and 9 and trypsinisation is not impaired. Digestion is conducted overnight at 37°C with 10–15 ng of MS‐grade Trypsin Gold (Promega) per µg of protein or per 10,000 cells, with continuous shaking at 400 rpm. Digestion is stopped by acidification with TFA (final concentration ∼2%).

Peptides are purified the next day. Microcolumns are prepared by punching five C18 disks [Empore SPE disks, CDS Analytical] and packing them into a 200‐µL pipette tip (also referred to as “StageTips” (stop‐and‐go‐extraction tips)) [7]. Each microcolumn is conditioned with 250 µL of LC‐MS grade MeCN, centrifuged at 3500–4000 RCF for 5 min, and the flow‐through is discarded. Conditioning continues with 250 µL of elution solvent (40% MeCN in H_2_O with 0.5% AcOH), followed by 250 µL of solvent A (H_2_O with 0.5% AcOH). Peptides are loaded onto the microcolumn and centrifuged, and the flow‐through is discarded. Peptides are then washed with 250 µL of solvent A and eluted with 40 µL of elution solvent in a new tube. The eluted peptides are dried in a HyperVac (VC2124, Gyrozen) at 2000 rpm at 30°C for at least 45 min. The peptides are then reconstituted in 10 µL solvent A per 1 µg of digested protein or 15,000 cells, ensuring standardized preparation for nanoflow LC‐MS/MS analysis. nLC‐MS/MS analysis is performed by injecting 100–400 ng peptides or an equivalent of 4500 cells (amount/cell count based on total protein content/cell count measured prior to digestion) onto an analytical column (Pepsep #1893477, 25 cm × 75 µm, 1.9 µm beads, C18 ReproSil AQ, Bruker GmbH, Mannheim, Germany). Peptides are eluted using a nanoElute 2 system (Bruker Daltonics GmbH) over 30 min. The gradient starts with 2% mobile phase B [0.5% AcOH (v/v) in 100% MeCN] and 98% mobile phase A [0.5% AcOH (v/v) in milliQ water], followed by an increase to 30% B over 22 min, a second increase to 95% B over 3 min and a plateau at 95% B for 5 min (flowrate of 300 nL/min). Acetic acid (AcOH) is preferred over traditional formic acid due to its ability to enhance ionization efficiency during electrospray ionization (ESI), thereby improving peptide detection sensitivity in bottom‐up proteomics workflows, as recently demonstrated [8, 9] and also confirmed in our own experiments using peptides from HeLa cells (Figure S1). Such an additive, with limited additional hydrophobicity compared to formic acid, is of particular interest for LC systems such as the nanoElute, which do not boast an additional pump for sample loading onto a trap column. In this case, the use of more hydrophobic, very efficient additive such as DMSO becomes impossible [10]. Of note, acetic acid is therefore also used in the solvents for StageTips (solvents A and B, mentioned above) to ensure consistency with the mobile phases. The separated peptides are ionized and sprayed into the timsTOF fleX mass spectrometer (Bruker Daltonics GmbH) through a 20‐µm ZDV Sprayer (Bruker Daltonics GmbH) using the CaptiveSpray source. Mass spectra are acquired in library‐free diaPASEF (Data‐Independent Acquisition‐Parallel Accumulation Serial Fragmentation) mode in an optimized mass range of *m/z* 300 to 1300 and an optimized mobility range of 0.64 to 1.45 V s/cm^2^, with a ramp time of 100 ms and duty cycle of 100% [11]. Collision energy settings were optimized from 28 eV at 1/K0 of 0.7 V s/cm^2^ to 50.5 eV at 1/K0 of 1.45 V s/cm^2^. The DIA window scheme followed a 3 × 8 pattern of 40 m/z widths covering the whole mass and mobility ranges and is shown in Table S2 and Figure S2.

In this study the raw data were analyzed in a spectrum‐centric way using FragPipe v22.0 [12], using quantification with DIA‐NN v1.9.0 [13], in library‐free mode, where diaTracer was used to build the spectral libraries through the generation of pseudo‐MS/MS spectra [14]. The search included a tolerance for up to four missed cleavages, peptide length ranging from 5 to 30 amino acids, and peptide mass ranging from 300 to 5000 Da. A 1% false discovery rate (FDR) was applied at the precursor, peptide, and protein group levels. For most cases, data were queried against the human or mouse reference proteome, as listed in Table 1. Exceptions included dental plaque and saliva proteomic data, which were queried against the human and the most commonly found oral bacterial proteomes [15], and the *Cistus creticus* seeds, where the *Gossypium barbadense* proteome was used, which is the closest species in terms of phylogeny. Up to two variable modifications were allowed per peptide. Variable modifications included oxidation of methionine residues and N‐terminal methionine excision, while carbamidomethylation of cysteine (arising from CAA alkylation) residues was set as a fixed modification. For BAL samples, analysis and visualization of quantitative proteomics data were done using FragPipe‐Analyst [16]. The general principles of the workflow followed are shown in Figure S3.

The general workflow was minimally adapted as needed for each sample type, with matrix‐specific details and deviations briefly described below (for details, see Table 1). As a general rule, the amount digested varied between matrices considering available amounts, preciousness of samples, need for future use, and expected profile variation at injection (some matrices tend to require a rerun with optimized injection volume to reach optimal peptide detection).

For CD138+ cell pellets (3000, 30,000, or 300,000 cells) and platelet samples, lysis was performed with 10–120 µL TFA, followed by neutralization with 6–8 volumes of 2 M tris base. For CD138+ samples, up to 30,000 lysed cells were transferred for digestion. For platelets, protein concentration was measured using a Nanodrop device (Quawell), and a volume corresponding to 2 µg of protein was transferred for digestion. Two platelet pellet freezing methods were compared (snap freezing in liquid nitrogen vs. direct freezing at −80°C) to assess their impact on the *ex vivo* proteomic profile. The detailed isolation protocols for CD138+ cells and platelets are provided in the supplementary material [17, 18, 19]. Dental plaque was collected as previously described [20]. *C. creticus* seeds were collected from the forest of Kaisariani, Greece, dried at room temperature for 10 days, saturated with water to assess water absorption capacity, and stored at −80°C. Prior to processing, seeds were pulverized on dry ice using a mortar and pestle, and 5 mg of seed powder is used per sample. For both the latter matrices, sample preparation followed the general TFA‐based lysis workflow, with two additional steps: (1) a 10‐min sonication during TFA lysis to enhance disruption, and (2) a post‐digestion clarification step using a 5‐min centrifugation at 4000 rpm through a single‐layer filter paper column (MN‐QF10, Macherey‐Nagel GmbH & Co., Düren, Germany) to remove particles.

BAL fluid [21] was processed by comparing four different protein precipitation methods: acetone, MeCN, ethanol, and the solid‐phase‐enhanced sample preparation (SP3) method [22]. Protein concentration was first measured using the Bradford assay. For each condition, a volume corresponding to 5 µg of protein was mixed with six volumes of organic solvent and incubated at 4°C for 1 h, followed by centrifugation at 18,200 RCF for 10 min at 4°C. Precipitated pellets are denatured with TFA and neutralized with 6–8 volumes of 2 M tris base. Protein precipitation from BAL fluid was also performed using the SP3 method [22]. Sera‐Mag A (GE45152105050250) and B (GE65152105050250) magnetic beads were removed from refrigeration, vortexed, and 20 µL each of A and B were combined with 160 µL of H_2_O. The tube was placed on a magnetic rack for 2 min to settle the beads, and the supernatant was discarded. The beads were rinsed three times with 200 µL of water. For precipitation, 5 µg of protein was transferred to a new tube, and 2 µL of beads and six volumes of MeCN were added, followed by incubation at 4°C for 18 min with shaking at 800 rpm. After immobilization on a magnetic rack for 2 min, the supernatant was discarded. The beads were washed twice with 80% ethanol and once with MeCN. For lysis, a digestion buffer was prepared by mixing 10 µL TFA with 90 µL tris base (2 M). Then 10 µL of TCEP/CAA was added for reduction‐alkylation of proteins at 37°C for 90 min. Finally, 360 µL H_2_O and 50 ng of trypsin were added to each sample, followed by overnight incubation at 37°C. The beads were immobilized on a magnetic rack for peptide elution and the eluate (∼470 µL) was first removed from the beads and kept aside. The beads were then resuspended in 50 µL of water containing 1% TFA, immobilized on a magnetic rack, and this eluate was also removed. Both eluants were combined for downstream analysis. The pH was adjusted with 2% TFA.

Saliva samples (200 µL), collected as described by Pappa et al. [23], and PPP extracts from CD138+ cells were processed via acetone precipitation. Saliva was treated with six volumes of acetone, and PPP with three volumes, followed by incubation at 4°C for 1 h and centrifugation at 18,200 RCF for 10 min at 4°C. Digestion proceeded as described in the general protocol above (details provided in Table 1). Murine heart tissue samples (NOD.Cg‐*Prkdc^scid^ Il2rg^1Wjl^* /SzJ male mice) were processed by grinding frozen samples on dry ice using a mortar and pestle. Tissue lysis was performed using 8 µL of low‐detergent RIPA buffer per mg of powdered tissue. Protein concentration was determined using the Lowry assay, and 4 µg of total protein were transferred for digestion. As the RIPA buffer does not require neutralization, samples proceed directly to reduction, alkylation, and digestion steps as described in the general workflow.

The lists of proteins detected in each matrix can be found in Tables S3–S10. The mass spectrometry proteomics data have been deposited in the ProteomeXchange Consortium via the PRIDE [24] partner repository with the dataset identifiers listed in Tables S3–S10. The coefficient of variation (CV) of each protein detected in all technical replicates was computed for all biological matrices to assess repeatability in protein quantification. The results demonstrated that most matrices exhibited low variability, with a significant proportion of protein groups achieving CV below 10% and 20% (Figure 1). For BAL samples, the different precipitation methods tested (acetone, MeCN, ethanol, and the SP3 method) performed differently. Acetone and MeCN precipitation emerged as the most effective methods (Figure S4), achieving the highest number of identified protein groups (average 1265 and 1221, respectively) and a precursor count of 12,408 and 11,572, respectively, with most protein groups displaying low variability. However, ethanol precipitation and the SP3 method were less effective and less robust, as they identified fewer protein groups, and exhibited broader CV distributions, suggesting reduced consistency in protein quantification. These results underscore the importance of precipitation method evaluation for specific matrices, as what could be considered similar approaches actually lead to very different results, significantly impacting protein recovery and repeatability. Based on these results, we chose acetone precipitation for the remaining sample types. While acetonitrile yielded slightly higher precursor identifications, acetone precipitation had the added advantage of producing pellets that were more easily solubilized in TFA. Previous studies have employed various approaches for BAL sample proteomics, including methanol:chloroform precipitation to remove lipids and surfactants [25]. However, this method was found to be inconsistent in contaminant removal, sometimes leading to interference in LC‐MS/MS analysis and requiring additional handling steps, such as molecular weight cutoff filtration and protein trapping using S‐Trap. Despite these efforts, reported proteome depth remained limited, with approximately 600 proteins identified. In contrast, the adapted SPEED protocol used here provides a simpler, faster, and safer alternative. The critical advantage of this method is the early addition of TFA, which significantly minimizes biological hazards as the acidic environment inactivates potential pathogens.

**FIGURE 1:**
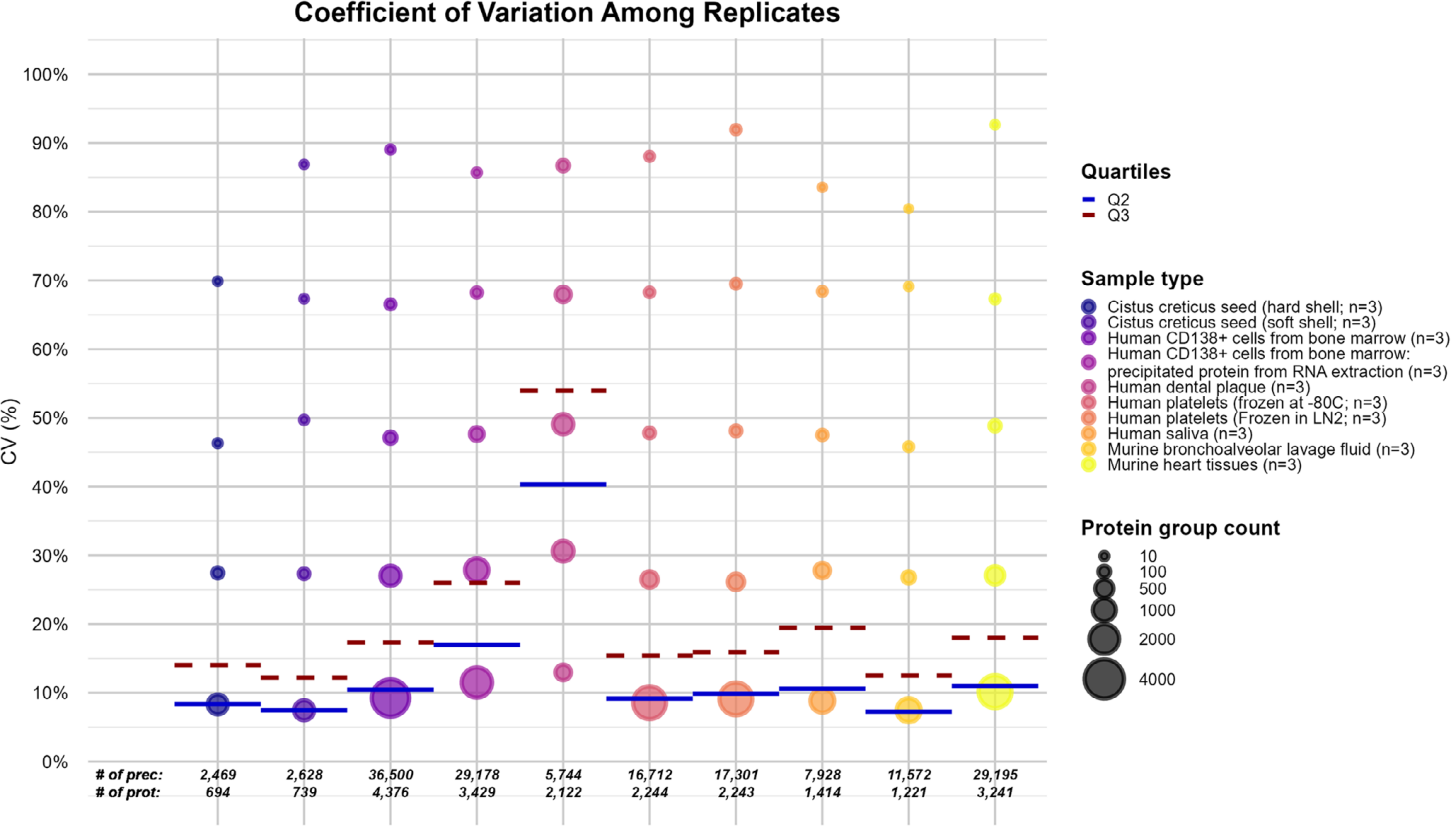
Number of protein groups detected and technical variability across different biological matrices. Plot displays the protein groups identified for each technical triplicate of the samples, grouped in five CV bins (vertically aligned bubbles positionned on the median CV of each bin) Higher coefficient of variation (CV) values are observed in low‐abundance or sparsely detected protein groups, while protein groups consistently identified across replicates exhibit lower CVs, indicating reliable quantification. LN2: liquid nitrogen.

CD138+ cells exhibited strong repeatability and good coverage, with the identification of up to 4376 proteins when injecting an equivalent of 4500 cells. For this analysis, we specifically selected the 30,000‐cell pellet, which provided the best results compared to the smaller and the larger ones (Figure S5). However, pellets of 3000 cells also provided decent coverage with 3839 proteins identified for injections of an equivalent of 1800 cells when searching across all files with different injection amounts (i.e., benefiting from match‐between‐runs), while searching only the three replicates of 1800 cells, as one would do if only these runs were available, resulted in the detection of ∼3600 proteins. When challenging our analytical pipeline to inject as low as 300 cells, we obtained close to 3300 proteins when benefiting from the match‐between runs, and 2302 proteins when using only these three specific replicates. This shows that our protocol enables the analysis of proteomes from specific cell fractions obtained from Fluorescence‐Activated Cell Sorting (FACS), for instance, where cell counts are often below 10,000. Additionally, 3429 proteins were identified in the protein phenol phase after RNA extraction of a CD138+ cell sample. This is a valuable finding, as it enables multiomics studies to be conducted from a single sample, maximizing data acquisition while preserving precious biological material. One should note that these repeatability tests are made on a single sample per matrix, aliquoted in several technical replicates, which means that there is no biological variation, as one would see in a cohort of samples. Therefore, the number of precursors and proteins detected is lower than what many experimental sample sets would provide, as pre‐processing software enriches their finding using match‐between‐runs features. Although we do not reach the >10,000 proteins reported in other studies [26], it is important to note that our workflow employs a single‐shot 30‐minute LC‐MS/MS gradient without any offline fractionation. In contrast, those protocols typically involve extensive pre‐fractionation into 12 fractions, each measured for 2 h, with one fraction run for 4 h, resulting in a total instrument time of approximately 28 h per sample [26]. The analysis of platelet pellets, frozen with liquid nitrogen or at −80°C, also demonstrated good repeatability, identifying around 2250 proteins despite the freezing method. Further evaluation revealed that samples frozen at −80°C showed better clustering in both the PCA and Pearson correlation matrix, indicating slightly higher consistency across replicates. Additionally, the median CV was marginally lower for these samples, further supporting their reproducibility. Overall, both freezing methods appear valid for platelet proteomics. However, to minimize technical bias, it is advisable to maintain consistency by using a single freezing approach within a given study. Additionally, freezing methods were tested within 3 months of platelet collection, and the authors cannot conclude on the freezing method for long‐term storage. Notably, while single‐shot analyses typically yield around 2000–2500 proteins, some studies employing extensive peptide fractionation have reported identifying up to 5000 proteins from platelet samples when several biologically different platelets were analyzed together [27].

*C. creticus* hard and soft seed samples exhibited higher variability compared to other matrices, with the identification of 694 and 739 proteins (using the *Gossypium barbadense* proteome as reference proteome), respectively, but still 75% of the proteins with CVs below 20% (hard shell). These results highlight the challenges inherent in seed proteomics, including the complex and resistant nature of seed matrices, which often impede protein extraction and digestion. Despite these difficulties, as noted in previous studies [28], we achieved significant proteome coverage using our adapted protocols, demonstrating the feasibility of proteomics in this challenging sample type. The biggest challenge in plant proteomics remains to access annotated protein sequences, as many species have not been sequenced. In our case, we used the *Gossypium barbadense* proteome, which is the closest species in terms of phylogeny. Therefore, no direct comparison with other studies can be made. This success underscores the potential for further refinement of seed proteomics workflows to enhance reproducibility and protein identifications, with applications in plant biology. But it also stresses the need to correctly plan such studies, including proteomics, only when protein sequences are available.

Dental plaque and saliva samples showed variable proteome profiles, with the identification of 2122 and 1414 proteins, respectively, and a broader distribution of CVs, especially for dental plaque samples. Our analysis of human saliva resulted in the identification of 1414 proteins across all samples, which is higher than the ∼1000 proteins reported in a recent study that employed a typical in‐solution digestion approach [29]. For dental plaque, we identified over 5000 unique peptides, which surpasses the ∼4500 peptides reported in a recent study using standard protocols [20]. This improved depth, coupled with a safer processing environment, is particularly relevant for handling oral samples with high microbial content. As for seeds, dental plaque is a hard and heterogeneous matrix, which may explain the higher heterogeneity observed in our data. Here we present the results from a sample collected from a male individual aged 44, with the plaque located on tooth number 36, which exhibited the best performance. Despite its liquid nature, saliva is also somewhat heterogeneous and viscous, making protein extraction more challenging. These results reflect the inherent difficulties associated with these sample types, which also host diverse microbial communities, significantly influencing their biological variability. The nature of these samples requires particular attention to biosafety and consistency during processing. The early addition of TFA in our protocol played a critical role in addressing these challenges by rapidly acidifying the samples, minimizing microbiological growth, and shearing potential viral DNA. This highlights the suitability of the workflow for microbiome‐rich and complex biological matrices, demonstrating its potential for applications in oral microbiology and related fields. Finally, murine heart tissues also exhibited strong repeatability, with 3241 proteins identified among three lysis technical replicates. Similar to our findings with CD138+ cells, we did not reach the >10,000 proteins reported in studies that employ extensive fractionation and longer gradients. For example, Giansanti et al. achieved deeper coverage using a 65‐min gradient combined with the measurement of four fractions [30], while a recent study by Mohanty et al. reported identifying ∼3,900 proteins using a 150‐min gradient. In contrast, our workflow employs a single‐shot 30‐min LC‐MS/MS analysis without the need for fractionation.

In summary, our adapted SPEED protocols demonstrate robust and reproducible proteomic data generation even from low‐input and challenging sample types, underscoring its versatility and practicality. The key novel aspects of this work include the streamlined use of TFA for direct protein denaturation across diverse biological matrices, notably dental plaque, *C. creticus* seeds, saliva, and platelets, which, to our knowledge, have not previously been lysed using TFA. This approach, combined with optimized neutralization and digestion conditions, ensures both enzymatic efficiency and biosafety. This approach simplifies sample handling without sacrificing depth of coverage, making it highly suitable for clinical and translational research, where sample quantity is often limited. Importantly, we provide practical guidance on which parameters are critical for successful application—such as maintaining tris concentration below 0.5 M prior to trypsin digestion to ensure optimal pH, and precise reduction and alkylation steps—while also identifying adjustable elements like input protein amounts and precipitation volumes that can be modified according to specific project needs. The introduction of a 30‐min LC gradient and careful optimization of DIA fragmentation windows further enhances throughput and proteome depth, leveraging the strengths of PASEF technology for scalable workflows.

Our study stresses the importance of conducting preliminary assessments of technical variability for each sample matrix, enabling researchers to identify limitations and adapt workflows accordingly, before designing larger experimental studies. By clearly distinguishing essential from flexible protocol parameters, we aimed at offering a reproducible and adaptable framework suitable for diverse bottom‐up proteomics studies, without compromising data quality. As proteomics gains wider interest, we strongly advocate for incorporating such minimal validation steps early in experimental design, to ensure that the research questions are appropriately addressed and that the studied biological variability can be optimally assessed. This approach not only optimizes study design and statistical power but also lays a solid foundation for generating meaningful biological insights.

## Conflicts of Interest

The authors declare no conflicts of interest.

## Supporting information




**Supporting Information file 1**: pmic70064‐sup‐0001‐SuppMat.docx

**Supporting Information file 2**: pmic70064‐sup‐0002‐Tables.xlsx

## Data Availability

Data are available via ProteomeXchange (keyword NKUAProt001) with unique identifiers PXD061640 [*Cistus creticus* seeds], PXD061569 [CD138+ cell pellets], PXD061575 [protein phenol phase of human CD138+ cells after RNA extraction], PXD061590 [human dental plaque], PXD061600 [human platelets], PXD061684 [human saliva], PXD061499 [murine bronchoalveolar lavage], PXD061636 [murine heart tissue] .
