# Supplementary material for: Easy Proteomics Sample Preparation: Technical Repeatability and Workflow Optimization Across 8 Biological Matrices in a New Core Facility Setting
Source: Proteomics. 2025 Oct 24;25(20):15–24. doi: 10.1002/pmic.70064 (PMC12576925; doi:10.1002/pmic.70064)
Supplement: Supplementary file 1 — Supporting Information file 1: pmic70064‐sup‐0001‐SuppMat.docx [file PMIC-25--s001.docx]

**Supporting information in relation to the manuscript entitled:**

**Enabling universal proteomics sample preparation: Technical repeatability and workflow optimisation across 10 biological matrices in a new core facility setting**

Paraskevi Karousi^1,2^, Maria Voumvouraki^2,3^, Panagiota Efstathia Nikolaou^4,5^, Ioannis Kollias^5^, Foteini Paradeisi^6^, Elena Sampanai^1^, Vasiliki Gkalea^7^, Ioannis Morianos^8^, Jerome Zoidakis^1,6^, Efstathios Kastritis^5^, Nikolaos Thomaidis^3^, Guillaume Médard^2,+^, Julie Courraud^2,5,+^

*^1^Section of Biochemistry and Molecular Biology, Department of Biology, School of Science, National and Kapodistrian University of Athens, Greece;*

*^2^Proteomics Core Facility, School of Science, National and Kapodistrian University of Athens, Greece;*

*^3^Laboratory of Analytical Chemistry, Department of Chemistry, School of Science, National & Kapodistrian University of Athens, Greece;*

*^4^Laboratory of Pharmacology, Department of Pharmacy, School of Health Sciences, National & Kapodistrian University of Athens, Greece;*

*^5^Section of Clinical Therapeutics, Department of Medicine, School of Health Sciences, National and Kapodistrian University of Athens, Greece;*

*^6^Proteomics Laboratory, Biomedical Research Foundation, Academy of Athens, Greece;*

*^7^Hematology Department, Alexandra General Hospital, Athens, Greece;*

*^8^Host Defense & Fungal Pathogenesis Lab, Institute of Molecular Biology and Biotechnology, Foundation for Research and Technology, Greece.*

+ Corresponding authors: Dr. Guillaume Médard, Proteomics Core Facility, School of Science, National and Kapodistrian University of Athens, 15701 Athens, Greece. E-mail: [gmedard@uoa.gr](mailto:gmedard@uoa.gr);

Dr. Julie Courraud, Proteomics Core Facility, School of Science, National and Kapodistrian University of Athens, 15701 Athens, Greece. E-mail: [jcourraud@uoa.gr](mailto:jcourraud@uoa.gr).

**Contents :**

- Figure S1. Comparison of protein sequence coverage between the usage of acetic or formic acid in the A and B mobile phases. Acetic acid provides higher sequence coverage and is therefore the preferable choice for improved proteomic analysis.
- Figure S2. DDA-PASEF distribution of HeLa >2+ tryptic peptide overlayed on chosen windows. The x-axis represents m/z (mass-to-charge ratio), while the y-axis represents 1/K0, which corresponds to ion mobility. The peptide distribution aligns well within the selected windows, confirming the appropriateness of the window selection for efficient sampling.
- Figure S3. Overview of the unified sample preparation workflow for bottom-up proteomics across diverse biological matrices.
- Figure S4. BAL samples were precipitated with acetone, MeCN, or ethanol, as well as with the SP3 method. MeCN and acetone precipitation yielded the best results regarding repeatability **(A)** and protein number identification **(B)**.
- Figure S5. Counts of detected precursors and proteins after injecting different numbers of CD138+ cells, derived from varying initial cell pellet counts. Good proteome coverage is achieved even with low cell numbers, demonstrating the downscalability of the method.
- CD138+ isolation protocol.
- Platelet isolation protocol.

**
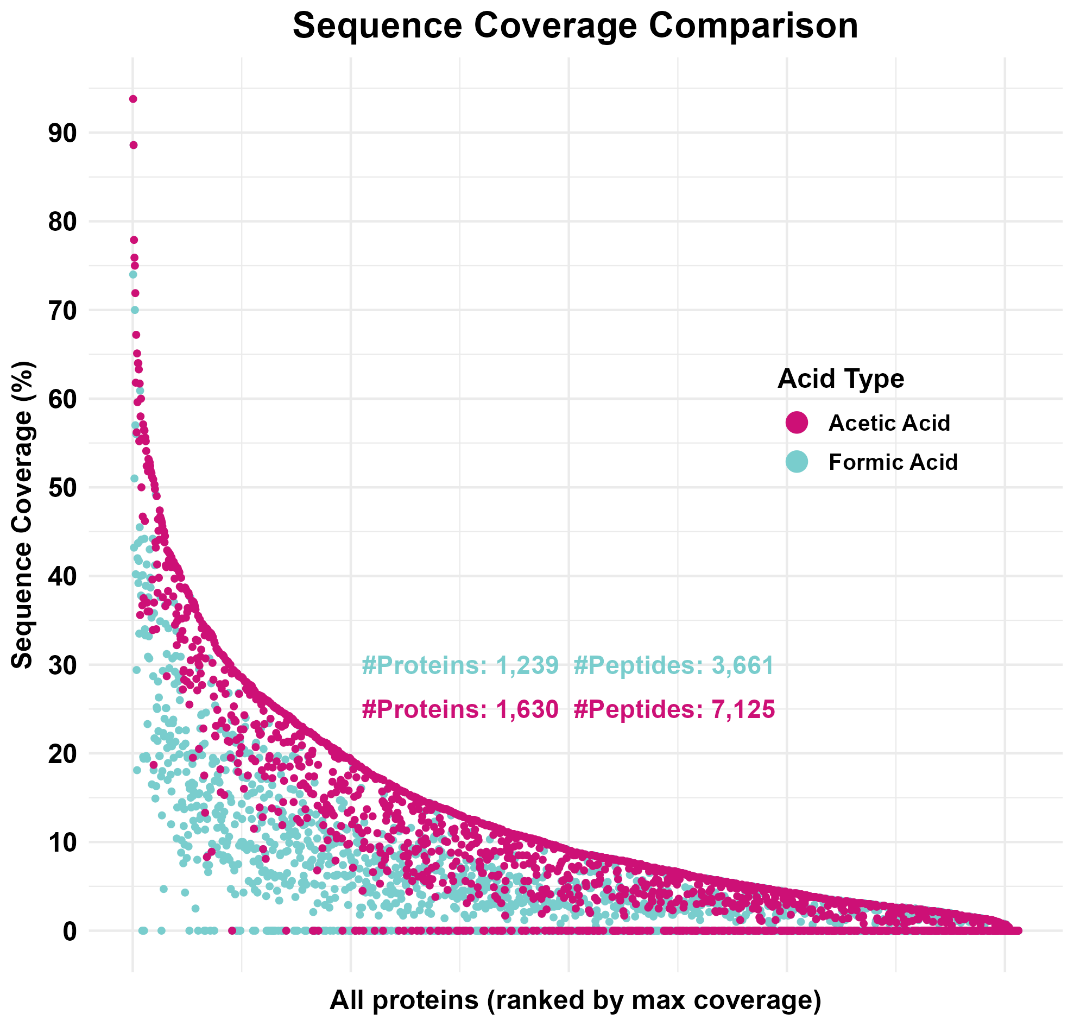
**

**Figure S1.** Comparison of protein sequence coverage between the usage of acetic or formic acid in the A and B mobile phases. Acetic acid provides higher sequence coverage and is therefore the preferable choice for improved proteomic analysis.

**
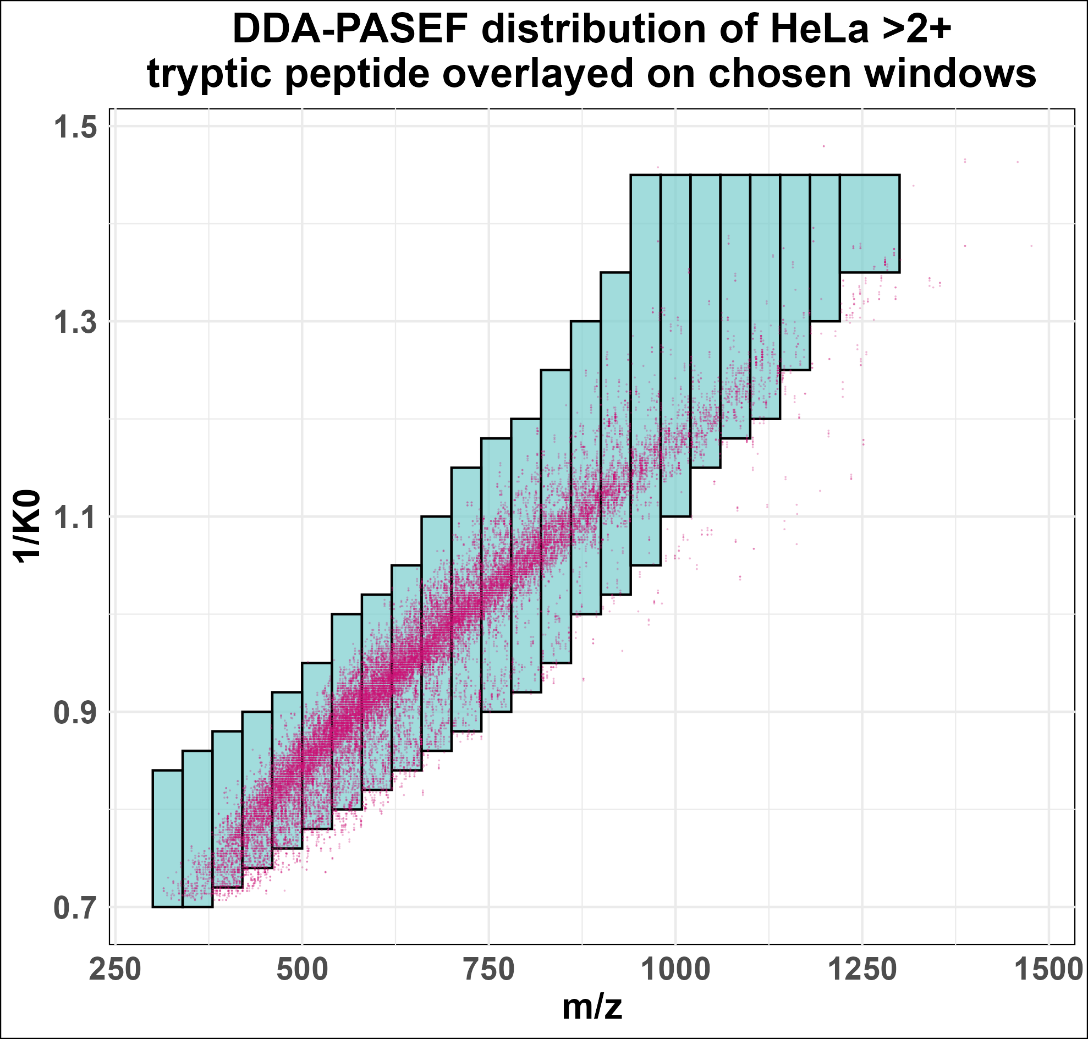
**

**Figure S2.** DDA-PASEF distribution of HeLa >2+ tryptic peptide overlayed on chosen windows. The x-axis represents m/z (mass-to-charge ratio), while the y-axis represents 1/K0, which corresponds to ion mobility. The peptide distribution aligns well within the selected windows, confirming the appropriateness of the window selection for efficient sampling.


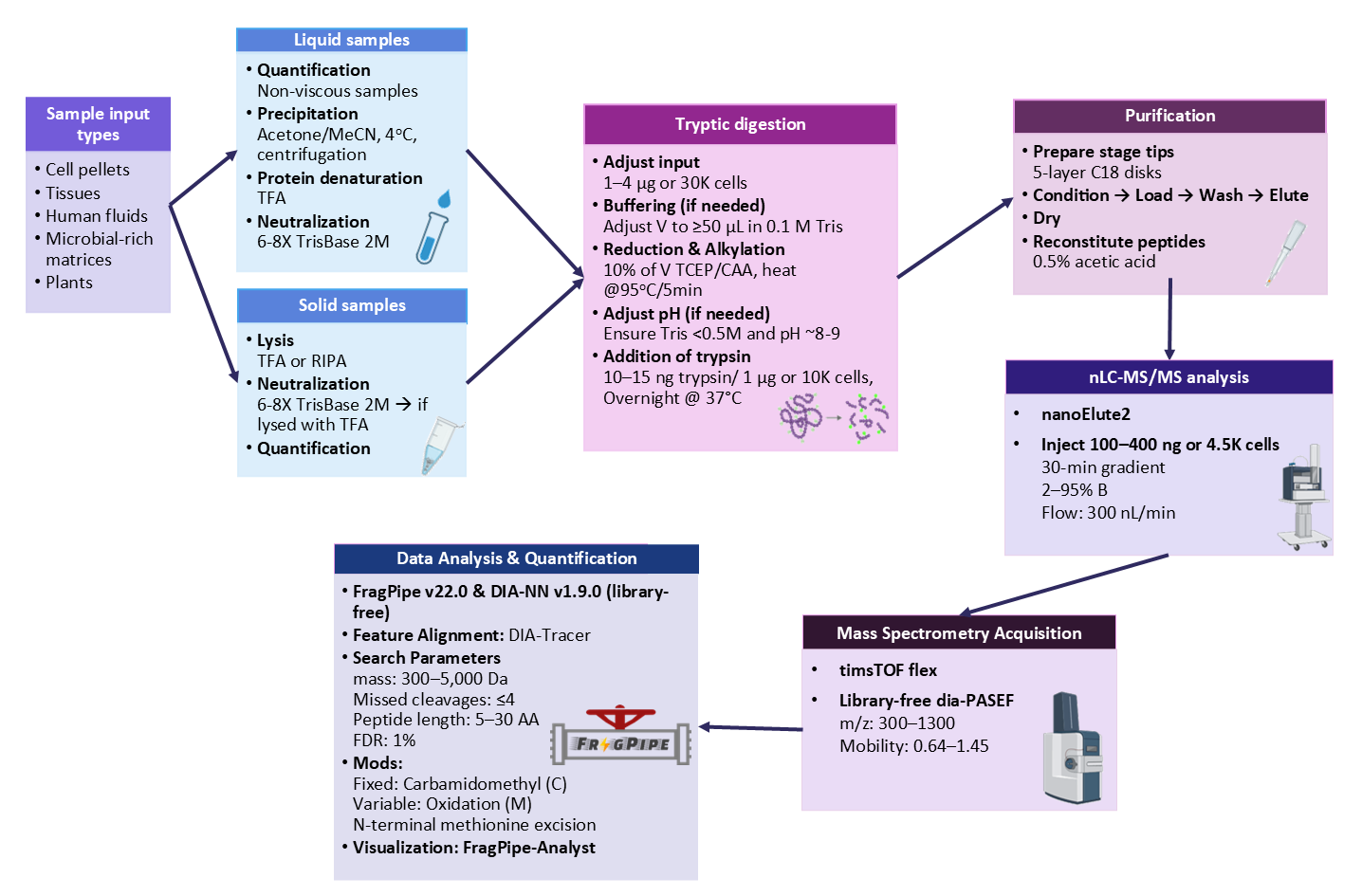


**Figure S3.** Overview of the unified sample preparation workflow for bottom-up proteomics across diverse biological matrices.

**
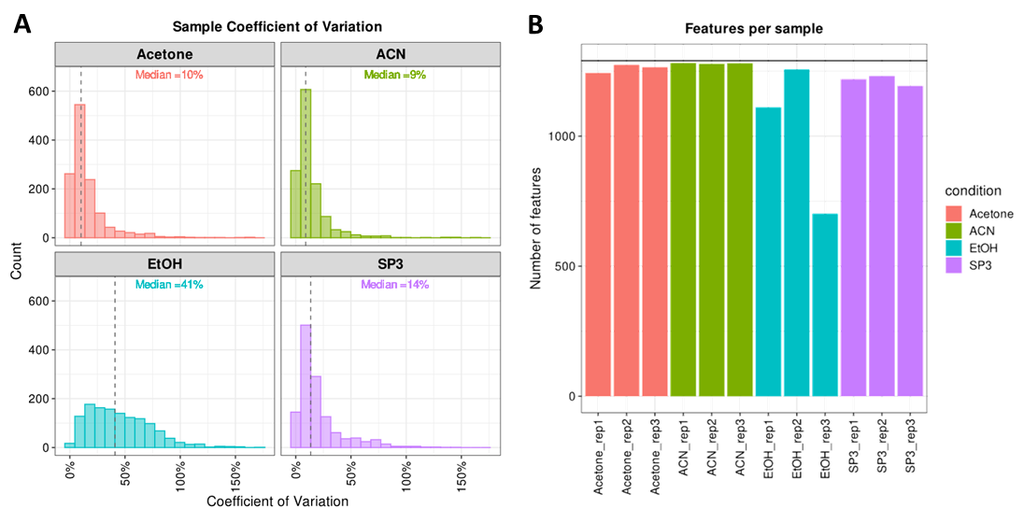
**

**Figure S4**. BAL samples were precipitated with acetone, MeCN, or ethanol, as well as with the SP3 method. MeCN and acetone precipitation yielded the best results regarding repeatability **(A)** and protein number identification **(B)**.

**
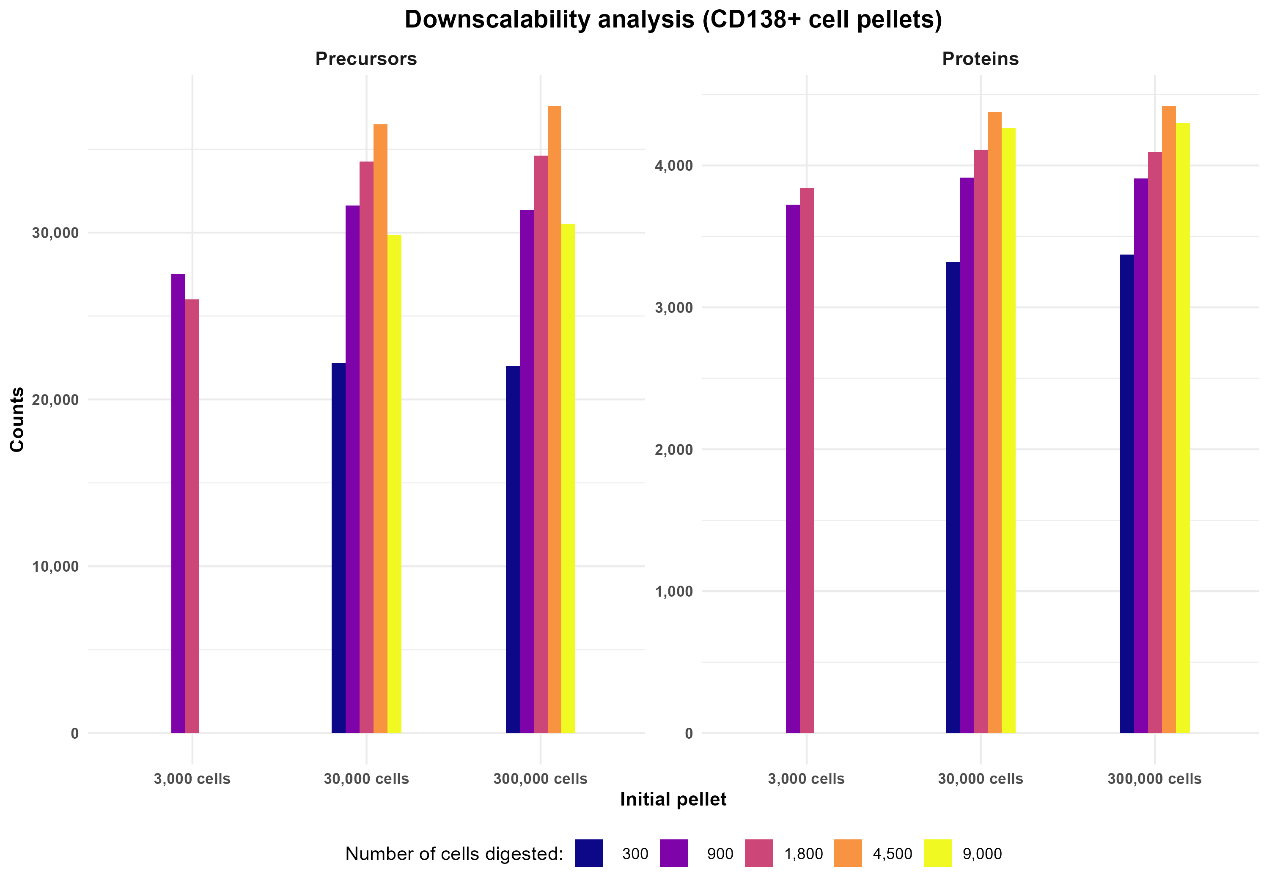
**

**Figure S5.** Counts of detected precursors and proteins after injecting different numbers of CD138+ cells, derived from varying initial cell pellet counts. Good proteome coverage is achieved even with low cell numbers, demonstrating the downscalability of the method.

**CD138+ isolation protocol**

**Immunomagnetic Separation of Cells Using the AutoMACS Pro System**

**(Miltenyi Biotec-adapted protocol)**

Immunomagnetic cell separation using the AutoMACS Pro system is an automated technique for separating specific cell types from heterogeneous mixtures. The principle utilizes antibody-coated paramagnetic beads that bind specifically to a target surface antigen. Cells of interest are separated from unbound cells using the AutoMACS Pro System magnet. Enriched cells of interest are suitable for downstream analyses such as flow cytometry, genomics and functional analysis.

**Materials**

- Countess II (Invitrogen)
- autoMACS Pro Separator (Miltenyi Biotec)
- autoMACS Column (Miltenyi Biotec)
- MACS SmartStrainers (100μm) (Miltenyi Biotec)
- Magnetic beads conjugated to specific antibodies (MicroBeads, Miltenyi Biotec)
- autoMACS Running Buffer (PBS, EDTA, BSA, 0.09% azide) (Miltenyi Biotec)
- autoMACS Washing Solution (contains detergent and stabilizer) (Miltenyi Biotec)
- Phosphate-buffered saline (PBS), pH 7.4
- Cell suspension containing target and non-target cells
- 15 mL, 50 mL falcon tubes
- Pipettes and sterile pipette tips

**Standard Operating Procedure from ficoll-derived BMMC (Manual Labeling)**

1. Count cells (Countess II, Invitrogen)
2. Centrifuge cell suspension at 400 x g for 10 min.
3. Discard supernatant and resuspend pellet in autoMACS Running Buffer (MACS Separation Buffer).

*Add running buffer to a final volume of 90μL for 10^7^ cells (adjust running buffer volume according to cell count. No more than 900μL running buffer is used for one separation).*

1. Add CD138 MicroBeads, human

*Add 10 μL microbeads for 10^7^ cells (adjust microbead amount according to cell count. No more than 100 μL microbeads is used for one separation).*

1. Pipet up-down to mix
2. Store for 15 min in the dark at 2-8^o^C.
3. Add Running Buffer to wash away unbound antibodies by centrifugation at 400 x g for 10 min. (max total volume of cell suspension = 5mL).
4. Discard the supernatant
5. Resuspend pellet in 2 mL running buffer.
6. The sample is ready for separation at the AutoMACS Pro System. For positive selection the ‘posseld’ protocol is selected. Isolated CD138+ cells and the rest of CD138- cells will be retrieved in different falcon tubes for downstream analysis.

**Standard Operating Procedure from total Bone Marrow Aspirate (WB Separation)**

1. Filter anticoagulated whole bone marrow aspirate (WBMA) (initially in EDTA tubes) using MACS SmartStrainers (100μm).
2. Add 500-1000 μL Running Buffer to dilute the suspension (~1/5 of the input WBMA volume)
3. Load filtered sample in AutoMACS Pro System. ‘Posselwb’ protocol is selected for selection from whole bone marrow aspirate. (Ab and buffer volumes are adjusted automatically)

Protocol adapted by [www.miltenyibiotec.com](http://www.miltenyibiotec.com)

**Platelet isolation protocol**

Blood was obtained using a 20-mL syringe with a 20-Gauge needle and was directly transferred into two 15-mL screw-capped centrifuge tubes containing 1 volume of Acid Citrate Dextrose (ACD) anticoagulant for 6 volumes of blood (total volume of 11.7 mL per tube). To prevent platelet activation, prostaglandin I2 (PGI2, Sigma-Aldrich, St. Louis, MO, USA) was added at a final concentration of 2 μg/mL [1]. The samples were centrifuged at ambient temperature for 20 min at 200 g without brake. Approximately 3.5 mL of supernatant platelet-rich plasma (PRP) was gently layered onto the surface of 5.4 mL of a continuous density gradient, as per the manufacturer's protocol (Sigma-Aldrich, St. Louis, MO, USA and centrifuged at 300 g for 20 min without brake [2]. The platelet suspension above the gradient was harvested and was centrifuged at 900 g for 10 min without brake (approximate volume of 10 mL). PGI2 was added at a final concentration of 0.3 μg/mL After removal of the supernatant, platelet pellets were washed once (800 g, 10 min, without brake) in 10 mL HEPES buffer (140 mM NaCl, 20 mM HEPES, pH 7.4) with addition of PGI2 at a final concentration of 0.3 μg/mL. Recovered platelets were resuspended in 1 mL HEPES buffer. To achieve additional depletion of residual leukocytes, CD45-labeled beads (Dyna beads, Invitrogen Dynal, Oslo, Norway) were used as per the manufacturer's protocol using a Dynamag^TM^-15 Magnet (Thermo Fisher Scientific, model: Dynamag^TM^-15, catalog number: 12301D [3]. Platelets were aliquoted and pelleted again (800 g, 10 min, without brake) with addition of PGI2 at a final concentration of 0.3 μg/mL. Platelet, erythrocyte and leukocyte count was performed using a Beckman Coulter DxH 900 Hematology Analyzer on the full blood and on the platelet suspension in HEPES buffer before aliquoting.

[1] Wrzyszcz A, Urbaniak J, Sapa A, Woźniak M. An efficient method for isolation of representative and contamination-free population of blood platelets for proteomic studies. Platelets 2017;28:43–53. https://doi.org/10.1080/09537104.2016.1209478.

[2] Ford TC, Graham J, Rickwood D. A new, rapid, one-step method for the isolation of platelets from human blood. Clinica Chimica Acta 1990;192:115–9. https://doi.org/10.1016/0009-8981(90)90075-4.

[3] Trichler SA, Bulla SC, Thomason J, Lunsford KV, Bulla C. Ultra-pure platelet isolation from canine whole blood. BMC Vet Res 2013;9:144. https://doi.org/10.1186/1746-6148-9-144.
